# Beyond Sex Differences: Body Composition and Dietary Behaviors

**DOI:** 10.3390/muscles4030038

**Published:** 2025-09-03

**Authors:** Cassandra Evans, Jaime Tartar, Jonathan Banks, Jennifer Austin, Jose Antonio

**Affiliations:** 1 Department of Health and Human Performance, Nova Southeastern University, Davie, FL 33328, USA; jose.antonio@nova.edu; 2 Department of Health Science, Rocky Mountain University of Health Professionals, Provo, UT 84606, USA; jennifer.austin@rm.edu; 3 Department of Psychology and Neuroscience, Nova Southeastern University, Davie, FL 33328, USA; tartar@nova.edu (J.T.);

**Keywords:** dietary behaviors, sex differences, body composition

## Abstract

Dietary behaviors influence nutrient intake and body composition, both of which are important determinants of an individual’s overall health. This study investigated sex differences in the associations between dietary behaviors and body composition. Using a cross-sectional design, adults completed the three-factor eating questionnaire (TFEQ-R18) and food cravings (FCI) to assess dietary behaviors. Body composition was assessed using bioelectrical impedance analysis. Relative to males, there were significantly higher levels of cognitive restraint and emotional eating in females. Males exhibited greater cravings for fatty foods and a higher frequency of acting on those cravings. Body fat percentage was positively correlated with emotional eating and cognitive restraint in both sexes. These results suggest that gender and body fat are key factors related to dietary behaviors.

## 1. Introduction

Dietary behaviors influence an individual’s health. Changes in body composition and body mass stem from imbalances, deficiencies, or excess intake. The types of food consumed can enhance health or further exacerbate health problems. Consumption of specific foods correlates with increased risks for noncommunicable diseases and obesity. Higher intakes of sugar, saturated fatty acids, and ultra-processed foods are linked to increased risks of cardiovascular disease [1,2]. High intakes of ultra-processed, energy-dense foods combined with low intakes of fiber and nutrient-dense foods are correlated with the development of chronic health conditions, including diabetes and metabolic syndrome [1,2]. The consumption of the aforementioned foods is related to an increase in body fat, another risk factor for noncommunicable diseases [3]. Conversely, lean body mass (LBM) is inversely related to mortality risk [4,5]. This suggests increases in LBM may be more advantageous for health outcomes compared to decreasing body fat alone.

LBM refers to muscles, bones, organs, connective tissue, and skeletal muscle [6]. Maintaining LBM is necessary for strength, mobility, and overall health. Dietary behaviors have a direct effect on body composition including muscle health. The preservation of LBM is especially relevant in the context of sarcopenia, aging, and chronic disease risk, where declines in muscle mass are linked to poorer health outcomes [7].

Dietary behaviors and anthropometrics are interrelated and mutually influential [8]. Dietary behaviors is a broad term that refers to the behavioral and psychological aspects of eating [9]. These can include constructs such as eating behaviors and food cravings. Numerous factors influence dietary intake, such as taste preferences, cultural influences, weight status, and sex [10,11,12]. A variety of eating behaviors have been linked to an individual’s dietary intake, including uncontrolled eating (UE), emotional eating (EE), and cognitive restraint (CR) [8]. Uncontrolled eating describes when an individual overeats, combined with not feeling in control of their intake. EE refers to an individual’s propensity to eat in response to negative emotional states. Cognitive restraint is the degree to which a person exerts behavioral control over their eating behavior, irrespective of their physiological hunger cues. On the other hand, food craving influences the type of foods consumed. A food craving is defined as “an intense desire to consume a particular food or food type that is difficult to resist” [13]. Food craving is not interchangeable with hunger. Food cravings may occur without hunger or the need for food [12]. Cravings experienced as the result of an environmental/external stimulus (ex., craving a hamburger after driving by a fast-food establishment) are cue-induced cravings. In contrast, tonic cravings occur in the absence of stimuli [12]. Most individuals will experience food cravings in their lifetime [12]. Food cravings are more prevalent among overweight/obese females compared to overweight/obese males [14]. Sex differences exist in craving type, frequency, intensity, and ability to regulate craving [12]. Females tend to score higher on cognitive restraint measures compared to males [15,16] and higher levels of cognitive restraint and emotional eating have been reported in females [16,17]. Increased food cravings (tonic) and dietary behaviors are associated with higher BMI and body mass [8,12].

Anthropometrics are frequently used to assess nutrition status and health in research and clinical settings. BMI, the ratio of weight to height, may not accurately provide information about an individual’s health. Thus, body composition, including body fat and lean body mass measurements, is better suited for assessing health status. Muscle and fat mass measurements, not BMI, are important for predicting mortality risk [18,19]. Sex differences in body composition exist in the general population. Males tend to have higher lean body mass (LBM) levels and bone mineral density (BMD) [20]. Females have relatively higher body fat levels, and the distribution of body fat differs from that of males due to hormones and for anatomical reasons (i.e., childbirth) [20,21,22]. Women tend to have higher levels of leptin, a hormone that regulates appetite, likely due to higher amounts of body fat [22]. Moreover, sex hormones affect eating behavior regarding food choices and total dietary intake [23,24].

Sex differences in dietary behaviors exist; however, the exact reason for this are unclear. Differences in body affect composition appetite regulation, hormonal responses, and energy needs [25]. It remains unclear if levels of body fat contribute to sex differences in dietary behaviors. The authors hypothesized that body fat percentage would be positively correlated with measures of dietary behaviors (uncontrolled eating, cognitive restraint, and emotional eating) and food cravings. Furthermore, the authors anticipated that males and females would have different dietary behaviors and food cravings. To our knowledge, few studies have explored sex differences in dietary behaviors when controlling for body fat and LBM. Thus the primary aim of this study was to examine the relationship between dietary behaviors and body composition. Given the variations in body composition between males and females, the secondary objective was to determine whether sex differences in dietary behaviors persist after controlling for body fat and LBM. Exploring the relationship between sex, body composition, and dietary behaviors will provide information about lifestyle practices that affect changes in body composition, including muscle maintenance or loss.

## 2. Results

Descriptive statistics for age, height, body mass, BMI, body fat, fat mass, LBM, and total physical activity for the week can be found in Table 1. All variables met the assumptions of normal distribution.

### 2.1. Sex Differences in Dietary Behaviors

Significant differences between males and females were found for two of the TFEQ-18 subscales: Cognitive Restraint (CR) and Emotional Eating (EE), as seen in Table 2. Specifically, females reported greater levels of emotional eating and greater levels of cognitive restraint. No significant differences were reported for uncontrolled eating (UE).

Significant sex differences were found in cravings for fat and the behavioral aspect of cravings for fats and fried foods, as seen in Table 2. Specifically, males reported higher cravings for fat and consumption of fats and fried foods than females. No significant differences were observed for other cravings (sweets, carbohydrates, fried foods) and the consumption of foods craved (sweets, carbohydrates, fried foods).

### 2.2. Pearson Correlations by Sex

In females, body fat was positively correlated with both cognitive restraint and emotional eating, whereas in males, body fat was only significantly correlated with emotional eating. Across both sexes, uncontrolled eating was strongly correlated with emotional eating, total food cravings, specific cravings for sweets, carbohydrates, and fats, as well as the consumption sweets in response to a craving. Among males, uncontrolled eating was related to fried food cravings and the behavioral component of total food cravings. In females, cognitive restraint was negatively correlated with all behavioral aspects of food cravings, while in males, it was only correlated with the behavioral aspect of fat cravings. Emotional eating was strongly linked to total food cravings and cravings for fats and sweets in both males and females. Additionally, in females only, emotional eating was significantly correlated with fried food cravings and the behavioral aspect of sweet cravings (Table 3).

### 2.3. Adjusted Sex Differences in Eating Behavior

To determine if the sex differences in eating behavior were still present after controlling for body fat, a series of regression analyses predicting dietary behavior outcomes from sex with body fat included as a covariate were performed. Consistent with our prior analyses, sex was a significant predictor, with females having lower scores for fat cravings (β = −0.38, R^2^ = 0.13, F(2, 171) = 12.33, *p* < 0.001) and the consumption of fat (β = −0.31, R^2^ = 0.08, F(2, 170) = 7.01, *p* = 0.001) and fried food (β = −0.38, R^2^ = 0.04, F(2, 171) = 3.22, *p* = 0.042) in response to respective cravings.

Counter to our prior analyses, in the model predicting emotional eating (R^2^ = 0.15, F(2, 171) = 15.62, *p* < 0.001), body fat was a significant predictor (b = 0.38, *p* < 0.001) but sex was no longer a predictor (b = 0.03, *p* = 0.766). The same was true for the model predicting cognitive restraint (R^2^ = 0.11, F(2, 171) = 10.32, *p* < 0.001): body fat was a significant predictor (b = 0.34, *p* < 0.001) but sex was no longer a predictor (b = −0.02, *p* = 0.782).

A simple linear regression showed LBM was a significant predictor for emotional eating (β = –0.43, R^2^ = 0.058, F(1, 172) = 10.55, *p* = 0.001), fat cravings (β = 0.01, R^2^ = 0.067, F(1, 172) = 12.31, *p* = 0.001), and the behavioral response to fat cravings (β = 0.01, R^2^ = 0.031, F(1, 171) = 5.49, *p* = 0.020). To determine if the sex differences in eating behavior were still present after controlling for LBM, a series of regression analyses predicting dietary behavior outcomes from sex with LBM included as a covariate were performed. Sex was a significant predictor in several models. Females reported lower fat cravings (β = −0.46, R^2^ = 0.13, F(2, 171) = 12.28, *p* < 0.001), lower fried food consumption (β = −0.47, R^2^ = 0.04, F(2, 171) = 3.40, *p* = 0.036), and lower fat consumption (β = −0.44, R^2^ = 0.08, F(2, 170) = 7.31, *p* = 0.001).Additionally, sex was a significant predictor of cognitive restraint (β = 15.34, R^2^ = 0.04, F(2, 171) = 3.69, *p* = 0.027).

## 3. Discussion

Understanding the relationship between body composition and sex on dietary behaviors is important for developing unique lifestyle interventions that promote healthy weight. The results of this study show that there are sex differences in dietary behaviors and food cravings.

### 3.1. Sex Differences in Dietary Behaviors

This study observed higher levels of emotional eating and cognitive restraint in females. These findings align with previous research which showed that females experience greater levels of emotional eating and cognitive restraint [26,27,28]. Women are more likely to use food to cope with emotions [29]. High-fat or sweet foods are commonly consumed when coping with negative emotions [30]. These types of foods are often calorically dense and activate part of the brain’s reward center (parahippocampal gyrus and anterior cingulated) [29,31]. Women may be more prone to cognitive restraint due to societal norms and the likelihood to engage in dieting behaviors [32]. Thus, women may use more restraint to offset caloric intakes from emotional eating.

Relative to females, males reported higher cravings for fat, fried foods and acting on respective cravings, as evidenced by independent t-tests (Table 2). Similarly to these findings, Zhou [33] and colleagues reported males having stronger desires for fats. It should be noted that the aforementioned study assessed food cravings using a visual analog scale postprandial which may affect craving scores due to levels of satiety. Other studies suggest that males tend to crave savory and fatty foods [27,34,35,36,37]. Compared to women, men are less knowledgeable about nutrition and make food choices based on what they enjoy rather than health [27,36,38,39]. Similarly, Nakumar et al. [40] theorize males are less concerned about calorie or fat intake, contributing to their preference for fatty foods. Nutritional goals and knowledge appear to contribute to preferences for fatty and savory foods.

Contrary to previously published studies, no differences in cravings for sweets were observed. The average scores for the reported cravings were <2, which corresponds to “rarely” on the FCI questionnaire. This suggests that cravings were low in both groups and aligns with previous research [41]. Several studies reported higher cravings for sweets in females, likely related to hormonal fluctuations and changes in mood [12,35,38,42,43,44]. Pelchat et al. [42] reported significantly higher sweet cravings in females. Interestingly these cravings declined with age, which suggests declining hormones, and childhood experiences with food influence food cravings as we age [42]. Females tend to experience more food cravings compared to men [45,46]. Conversely, our findings observed no differences in total food craving scores. Other factors besides sex such as physical activity or dietary intake influence food cravings [44,47,48]. Studies have reported correlations between unhealthy eating behaviors and higher BMIs as well as sedentary behaviors [49,50,51]. The current study did not examine the relationship between physical activity and food cravings or assess dietary intake. These may account for the differences observed in this investigation.

### 3.2. Pearson Correlations by Sex

The relationship between body fat and emotional eating suggests that individuals with higher body fat are more likely to eat in response to negative emotional states. Foods that are hyper palatable and calorically dense are typically consumed during emotional eating [29,31,52]. Over time, consumption of calorically dense foods contributes to increases in body fat via excess calorie intake. Among females, body fat was associated with greater cognitive restraint. This relationship may be the result of rigid dietary restraint. Although cognitive restraint and emotional eating may appear contradictory, they often coexist. Individuals who engage in rigid forms of restraint may experience increased cravings due to chronic restriction [53,54]. This pattern reflects a cyclical relationship where attempts to strictly limit intake of specific foods leads to subsequent episodes of disinhibited eating or binge eating when restraint is absent [54]. Polivy and Herman (2020) [53] theorize that cognitive restriction increases the reward of “forbidden” foods. This increases the likelihood of overeating in response to emotional distress, food cues, or lapses in self-restraint. The cyclical pattern of restriction followed by overeating may contribute to weight gain and increased body fat over time.

In females, uncontrolled eating was positively correlated with emotional eating, as well as cravings for sweets, fats, fried foods, and the total food craving score. A similar relationship was observed in males, where uncontrolled eating was positively associated with emotional eating, total food cravings, specific cravings for fats, sweets, carbohydrates, and fried foods, as well as behavioral engagement of cravings (total FCI behavior), particularly for sweets. Emotional eating occurs independently of hunger and lowers inhibitory control to foods cues. Combined with an increase in reactivity to food cues, emotional eating increases the susceptibility to uncontrolled eating [55]. Using food as a coping mechanism elevates caloric intake and may be perceived as a loss of control. This effect is particularly pronounced among females, who are more attuned to dietary restraint, health, and appearance concerns [32]. The relationship between uncontrolled eating and emotional eating suggests the combination of reduced inhibitory control and increased sensitivity to food cues increase food cravings.

Cognitive restraint was negatively correlated with all behavioral aspects of food cravings, suggesting that women with higher restraint actively limit their intake despite experiencing cravings. Conversely, cognitive restraint was only associated with consumptions of fats in males. Women often exhibit greater dietary restraint and are more likely to choose foods based on their calorie content [32,37,56,57]. These sex-specific patterns may reflect greater internalization of dietary norms and health or appearance-related goals in females [32,56]. The absence of restraint-related correlations and the presence of strong links between cravings and behavioral follow-through for multiple food categories support the notion that men are more likely to eat for pleasure.

Emotional eating was strongly associated with total food cravings and cravings for fats and sweets in both males and females. This aligns with the existing literature suggesting that highly palatable foods are often consumed for their perceived “comforting” effect in response to negative emotional states [29,31,52]. Individuals with high levels of emotional eating often report stronger cravings for energy-dense foods, particularly those rich in fat [52,58]. In females only, emotional eating was significantly correlated with fried food cravings and the behavioral aspect of sweet cravings. Hormonal fluctuations throughout the month affect mood and increase cravings for high-fat and highly sweet foods in women [23,24,59]. The effect of hormones may explain the sex-specific differences in emotional eating observed in this study; however, female menstrual cycles were not assessed in this study. In females, LBM was inversely correlated with cravings for fried foods and the consumption of sweets in response to sweet cravings. To our knowledge, there is no literature that examines the relationship between LBM and food cravings. Given the relationship between body fat and other dietary behaviors, the inverse relationship with LBM is plausible. More research is needed to determine if LBM influences dietary behaviors and any underlying mechanisms.

### 3.3. Adjusted Sex Differences in Dietary Behaviors

After controlling for lean body mass, sex remained a significant predictor of several craving-related outcomes and eating behaviors, with females reporting lower fat cravings, consumption of fried food, consumption of fat, and higher cognitive restraint. To the authors’ knowledge, this is the first study to examine if any relationship between LBM and dietary behaviors exists. After controlling for body fat, sex remained a significant predictor of some aspects of cravings and behavior, with males exhibiting higher levels of fat cravings and the behavioral aspects of fat and fried food cravings. However, our findings suggest sex differences in some dietary behaviors may be confounded by differences in body composition. Specifically, body fat remained a significant predictor, irrespective of sex, for cognitive restraint and emotional eating. Our findings suggest that body fat, rather than LBM, is more consistently associated with emotional eating and cognitive restraint. While our study relied on bioimpedance body composition measures, the findings are aligned with prior research which shows a relationship between the BMI and higher scores of conscious restraints and emotional eating [15,49,51]. Additionally, BMI is related to higher levels of overconsumption and uncontrolled eating [15,60]. Emotional eating is often accompanied by overeating and occurs more often in overweight or obese individuals. Despite the average BMI score of 24.74 (normal weight 18.5–24.9), body fat was still a predictor and positively correlated with emotional eating. The participant’s stress levels were not assessed in the current study, therefore its effects on emotional eating cannot be determined. Several studies report a positive correlation between body fat and CR, which supports the findings of this investigation [15]. It has been theorized that individuals who engaged in high levels of restraint may engage in more problematic eating behaviors such as emotional eating, potentially leading to increases in body fat and/or body mass [61]. Conversely, Boschi et al. [62] reported lower levels of restraint in obese subjects compared to normal and overweight subjects. Stewart et al. [63] suggests that caloric restriction and dietary restraint, as often occur during fad diets, may lead to unintentional overeating. Participants in the current study were not engaged in weight loss, so it is less likely that the current findings were due to attempts at caloric restriction resulting from dietary restraint. Westenhoefer et al. [54] suggest that there are two types of dietary restraint: rigid or more flexible. Rigidity is generally associated with less favorable outcomes. It remains unclear whether individuals have increased their cravings as a result of higher body fat, or whether stronger cravings and related eating behaviors contribute to body fat accumulation.

Our study has some limitations. Although validated surveys were used, it still relies on self-reported data. Self-reported dietary data are often susceptible to recall bias, underreporting, and social desirability effects. Some of the foods listed in the FCI are animal-based and may not accurately assess food cravings in the vegetarian population. Mood and stress levels were not assessed in this study. Negative emotions are associated in changes in dietary behaviors [30,58]. Mood and stress levels were not assessed in this study and their effects on dietary behaviors cannot be ruled out. Fluctuating hormones during the menstrual cycle affect appetite [23,24,59]. Menstrual cycles were not assessed for female participants, thus the effects of hormones on food cravings and eating behaviors cannot be ruled out. Although no upper age limit was set for the current study, most of the participants were college students. Yun et al. [25] suggests the dietary intake of students is characterized by consumption of calorically dense foods with minimal intake of fruits and vegetables, which may influence the results. Thus, it is possible that the inclusion of a majority-college-student sample may have altered these findings. Future studies should include the assessment of dietary intakes.

## 4. Materials and Methods

This study used a cross-sectional design to examine the relationships between dietary behaviors and body composition in adult male (age 21.73 ± 5.23; *n* = 63) and female participants (age 20.7 ± 3.95; *n* = 112). Subjects were recruited from the Nova Southeastern University campus located in Davie, Florida. Individuals were excluded if they had a history of eating disorders or were actively engaged in weight loss efforts. Exclusion criteria were assessed via self-report. All participants underwent an informed consent process in accordance with the Declaration of Helsinki and an approved IRB protocol submitted to the institutional review board of Nova Southeastern University and Rocky Mountain University of Health Professionals. Subjects were instructed to arrive at the laboratory following their usual daily routine. During the testing session, subjects underwent testing for body composition and completed an online survey consisting of demographic questions, TFEQ-18, and FCI.

### 4.1. Anthropometrics

Body composition was assessed using a multi-frequency bioelectrical impedance device (InBody^®®^). The InBody bioelectrical impedance analyzer (BIA) is a validated and reliable tool commonly used in research settings to assess body composition, including fat mass, lean body mass, and total body water [64]. Participants were instructed to abstain from food, liquids, and strenuous activity for at least 4 h prior to testing to minimize measurement error. Individuals who were pregnant or who had pacemakers or other implanted electronic devices were excluded for safety and accuracy. Study participants stood on the device’s platform barefoot with the soles of their feet on the electrodes and then grasped the unit’s handles with their thumb and fingers to maintain direct contact with the electrodes. They stood still for ~1 min with their elbows fully extended and their shoulder joints at a ~30-degree angle.

### 4.2. Dietary Behaviors

Dietary behaviors were assessed using two questionnaires: the Three Factor Eating Questionnaire (TFEQ-18) and the Food Craving Inventory (FCI).

The TFEQ-R18 consists of 18 questions assessing cognitive restraint, emotional eating, and uncontrolled eating. The instrument is a shortened and revised version of the original 51-item TFEQ and considered both valid and reliable [65]. Responses to each of the 18 items are scored between 1 and 4 (definitely true/mostly true/mostly false/definitely false), and item scores are summed into scale scores for cognitive restraint, uncontrolled eating, and emotional eating. Raw scale scores are converted to a 0–100 scale as previously described by de Lauzon et al. [65].

The FCI is a previously validated self-reported survey assessing general and specific food cravings. This assessment differs from other assessments as it gathers information about specific foods rather than an individual’s subjective experience. Participants are asked about cravings for specific foods over the last month. A follow-up question asks how often they consumed the craved food as a measure of consumption [66]. Answers are recorded using a Likert scale (1(never)–5 (always/almost every time). The measure has four subscales; fried foods, dietary fats, sweets, and starches [13]. The average of each subscale is calculated and higher score on a subscale or total score indicates a greater frequency of craving for that category of food.

### 4.3. Statistical Analysis

All statistical analyses were conducted using R version 4.5.0 (2025-04-11). Descriptive statistics were computed for all variables. A two-tailed independent samples t-test was conducted to examine sex differences in dietary outcomes and body composition. Welch’s t-test was employed for all comparisons by sex, as it does not assume equal variances or sample sizes [67,68]. The significance level was at *p* < 0.05, and all t-tests used a two-tailed *p*-value. A Pearson correlation was conducted to assess any relationships between body fat percentage and dietary behavior outcomes prior to multivariable analysis. Linear regression analyses were performed to examine whether sex predicted dietary behavior outcomes after controlling for body fat percentage.

## 5. Conclusions

The results of this study demonstrate the complex interplay between sex, body composition, and dietary behaviors. Males exhibited greater cravings for fatty foods, while females had higher levels of emotional eating and cognitive restraint. However, after controlling for sex, body fat was a stronger predictor for emotional eating and cognitive restraint. The relationship between body composition and eating behaviors suggests that physiological factors may play a role in eating patterns. Both sexes craved highly palatable foods (fats, sweets, fried foods) related to emotional eating. Men were more likely to act on those cravings suggesting more hedonic, less restrained eating. Females experienced cravings but actively inhibited behavioral responses due to higher levels of cognitive restraint. These findings suggest that sex-specific differences in the types of foods craved exist. However, dietary behaviors such as cognitive restraint and emotional eating are more related to body composition than sex. These findings may have practical implications for creating individualized nutrition recommendations that help support healthy behaviors. Future research should further investigate whether certain behaviors and cravings lead to excess body fat over time or higher body fat contributes to increased cravings and emotional eating.

## Figures and Tables

**Table 1:** Participant characteristics.

Variable	Total		Male (*n* = 63)		Female (*n* = 112)	
Age (yrs)	21.07 ±	4.465	21.73 ±	5.23	20.7 ±	3.95
Height (cm)	169.84 ±	10.22	179.557 ±	7.79	164.375 ±	6.79
Body mass (kg)	71.03 ±	16.46	82.203 ±	13.93	64.752 ±	14.32
BMI	24.74 ±	4.29	25.487 ±	3.71	24.321 ±	4.54
Body Fat (%)	24.58 ±	10.03	16.951 ±	7.26	28.872 ±	8.76
Fat Mass (kg)	17.85 ±	8.97	14.433 ±	7.78	19.777 ±	9.05
LBM (kg)	53.77 ±	13.33	67.762 ±	9.85	45.905 ±	7.12

**Table 2:** Sex differences in dietary behaviors.

	Male (*n* = 62)		Female (*n* = 112)				
	Mean	Std. Deviation	Mean	Std. Deviation	t Value	Equal 95% Confidence Interval (Lower, Upper)	*p*-Value
UE	50.776	19.177	48.082	16.270	0.935	(−3.01, 8.40)	0.352
CR	43.638	20.216	51.924	24.736	−2.387	(−15.15, −1.42)	0.018 *
EE	18.638	21.835	30.455	23.665	−3.317	(−18.86, −4.77)	0.001 *
FCI Craving Scales							
FCI (total)	9.753	2.578	9.058	1.735	1.898	(−0.03, 1.42)	0.061
Fat	2.241	0.636	1.827	0.459	4.516	(0.23, 0.59)	<0.001 *
Sweets	2.347	0.836	2.417	0.705	−0.564	(−0.32, 0.18)	0.574
Carbohydrates	2.379	0.792	2.232	0.583	1.283	(−0.08, 0.37)	0.203
Fried Foods	2.786	0.827	2.582	0.639	1.688	(−0.03, 0.44)	0.095
FCI Behavioral Scales							
FCI (total)	8.514	3.084	7.911	2.001	1.393	(−0.26, 1.46)	0.167
Fat	1.963	0.698	1.627	0.483	3.355	(0.14, 0.54)	0.001 *
Sweets	2.012	0.872	2.031	0.634	−0.156	(−0.27, 0.23)	0.876
Carbohydrates	2.162	0.753	1.960	0.624	1.779	(−0.02, 0.42)	0.078
Fried Foods	2.581	0.913	2.292	0.724	2.141	(0.02, 0.55)	0.035 *

* *p* < 0.05, indicating statistical significance.

**Table 3:** Correlations among study measures by sex (data for females are presented above the diagonal and males presented below the diagonal).

	BodyFat	LBM	UE	CR	EE	FCI	Fat	Sweet	CHO	FriedFood	FCI_B	Fat_B	Sweet_B	CHO_B	Fried_B
BodyFat	--	−0.036	0.055	**0.305**	**0.309**	−0.051	−0.053	−0.124	−0.078	0.108	−0.064	−0.088	−0.103	−0.115	0.072
LBM	0.102	--	0.067	0.117	−0.131	−0.127	−0.022	−0.172	0.059	** −0.194 **	−0.132	−0.099	** −0.217 **	0.066	−0.168
UE	0.158	0.076	--	0.114	**0.632**	**0.305**	**0.193**	**0.254**	**0.248**	0.184	0.129	0.094	**0.186**	0.099	0.046
CR	0.231	0.12	0.173	--	0.163	−0.066	−0.081	−0.002	−0.054	−0.070	**−0.326**	**−0.257**	**−0.302**	**−0.261**	**−0.244**
EE	**0.349**	−0.026	**0.498**	0.071	--	**0.229**	0.093	**0.242**	0.102	**0.195**	0.156	0.050	**0.266**	0.020	0.150
FCI	−0.038	−0.028	**0.423**	−0.155	**0.254**	--	**0.703**	**0.747**	**0.718**	**0.731**	**0.646**	**0.446**	**0.618**	**0.499**	**0.524**
Fat	−0.024	−0.045	**0.368**	−0.228	**0.266**	**0.876**	--	**0.329**	**0.425**	**0.438**	**0.618**	**0.746**	**0.432**	**0.428**	**0.469**
Sweet	−0.062	0.129	**0.42**	−0.082	**0.286**	**0.835**	**0.691**	--	**0.376**	**0.346**	**0.349**	0.142	**0.628**	0.166	0.180
CHO	−0.073	−0.101	**0.34**	−0.072	0.159	**0.841**	**0.704**	**0.583**	--	**0.317**	**0.468**	**0.292**	**0.32**	**0.705**	**0.218**
FriedFood	0.031	−0.088	**0.287**	−0.158	0.146	**0.794**	**0.59**	**0.502**	**0.535**	--	**0.499**	**0.252**	**0.382**	**0.223**	**0.689**
FCI_B	0.066	−0.038	**0.27**	−0.247	0.177	**0.756**	**0.681**	**0.542**	**0.66**	**0.653**	--	**0.816**	**0.815**	**0.785**	**0.84**
Fat_B	0.044	−0.037	0.184	**−0.317**	0.201	**0.598**	**0.681**	**0.369**	**0.492**	**0.475**	**0.906**	--	**0.56**	**0.615**	**0.576**
Sweet_B	0.045	0.137	**0.296**	−0.218	0.189	**0.689**	**0.607**	**0.71**	**0.489**	**0.495**	**0.868**	**0.756**	--	**0.477**	**0.6**
CHO_B	−0.080	−0.171	0.161	−0.110	0.109	**0.703**	**0.608**	**0.425**	**0.838**	**0.467**	**0.828**	**0.693**	**0.596**	--	**0.489**
Fried_B	0.109	−0.048	0.226	−0.226	0.115	**0.602**	**0.465**	**0.325**	**0.475**	**0.737**	**0.885**	**0.737**	**0.674**	**0.631**	--

Note: all correlations presented in bold are significant at the *p* > 0.05 level. UE—uncontrolled eating, CR—cognitive restraint, EE—emotional eating, FIC—food craving inventory score, CHO—carbohydrate, _B denotes behavioral aspects of respective craving.

## Data Availability

Further inquiries can be directed to the corresponding author.
